# Improving the Interfacial Stability between Lithium and Solid‐State Electrolyte via Dipole‐Structured Lithium Layer Deposited on Graphene Oxide

**DOI:** 10.1002/advs.202000237

**Published:** 2020-05-18

**Authors:** Muqin Wang, Zhe Peng, Wenwei Luo, Qiang Zhang, Zhendong Li, Yun Zhu, Huan Lin, Liangting Cai, Xiayin Yao, Chuying Ouyang, Deyu Wang

**Affiliations:** ^1^ Ningbo Institute of Materials Technology and Engineering Chinese Academy of Sciences Ningbo 315201 China; ^2^ Department of Physics Laboratory of Computational Materials Physics Jiangxi Normal University Nanchang 330022 China

**Keywords:** dipole structure, graphene oxide, lithium metal anodes, lithium metal batteries, solid electrolyte interfaces

## Abstract

Utilization of lithium (Li) metal anode in solid‐state batteries (SSBs) with sulfide solid‐state electrolyte (SSE) is hindered by the instable Li/SSE interface. A general solution to solve this problem is to place an expensive indium (In) foil between the SSE and Li, while it decreases the output voltage and thus the energy density of the battery. In this work, an alternative strategy is demonstrated to boost the cycling performances of SSB by wrapping a graphene oxide (GO) layer on the anode. According to density functional theory results, initial deposition of a thin Li layer on the defective GO sheets leads to the formation of a dipole structure, due to the electron‐withdrawing ability of GO acting on Li. By incorporating GO sheets in a nanocomposite of copper‐cuprous oxide‐GO (Cu‐Cu_2_O‐GO, CCG), a composite Li anode enables a high coulombic efficiency above 99.5% over 120 cycles for an SSB using Li_10_GeP_2_S_12_ SSE and LiCoO_2_ cathode, and the sulfide SSE is not chemically decomposed after cycling. The highest occupied molecule orbital/lowest unoccupied molecular orbital energy gap of this Li/GO dipole structure likely stretches over those of Li and sulfide SSE, enabling stabilized Li/SSE interface that can replace the expensive In layer as Li protective structure in SSBs.

## Graphical abstract

A dipole‐structured Li‐GO interface can be achieved during initial Li plating on graphene oxide sheet to provide a favorable energy band change with lowered reactivity of Li metal. Uniform Li deposition with stabilized Li/electrolyte can be achieved for significantly improved cycling stability of all solid‐state batteries using sulfide electrolyte.

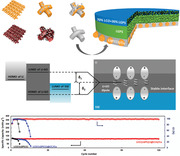

The state‐of‐the‐art lithium (Li)‐ion batteries using graphite anodes (372 mAh g^−1^ theoretical specific capacity) are approaching their upper energy density limit ≈300 Wh kg^−1^, which falls short of expectations for the energy storage systems.^[^ ^1^ ^]^ Li metal is one of the most promising anode materials for next‐generation high‐energy‐density batteries due to its ultrahigh specific capacity of 3860 mAh g^−1^ and the lowest redox potential of −3.04 V (vs standard hydrogen electrode).^[^ ^1^, ^2^, ^3^ ^]^ However, the practical application of lithium metal batteries (LMBs) is stagnated due to the poor cycling stability of Li metal anode.^[^ ^4^, ^5^ ^]^ The instability of Li metal anode is mainly caused by the aggressive side reactions between reactive Li and liquid organic electrolytes, whereas the highly generated mossy/dendritic microstructures on Li surfaces seriously accelerate the consumption of active Li and electrolytes.^[^ ^6^, ^7^, ^8^ ^]^ Moreover, thermal runaway inducing fire and explosion makes the LMBs using liquid organic electrolytes unsafe in hazardous conditions.^[^ ^9^, ^10^ ^]^ A plenty of efforts have been made to address the unstable Li/electrolyte interfaces, including the formulation of alternative electrolytes,^[^ ^11^, ^12^, ^13^, ^14^ ^]^ artificial protective layers,^[^ ^15^, ^16^, ^17^ ^]^ and 3D‐structured electrodes,^[^ ^18^, ^19^, ^20^ ^]^ showing considerable ability of Li metal protection.

An alternative pathway to address the safety issue of LMBs in keeping their high‐energy‐density relies on the use of solid‐state electrolytes (SSEs) instead of inflammable organic liquids, whereas the as‐generated solid‐state batteries (SSBs) are highly studied in recent years.^[^ ^21^, ^22^, ^23^ ^]^ Nevertheless, besides the achievement of comparable Li^+^ ionic conductivity at room temperature, the instability of Li/electrolyte interface persists prior to the practical application of SSBs.^[^ ^24^, ^25^, ^26^ ^]^ Most of the available SSEs react chemically with Li metal once in contact due to their unmatched highest occupied molecule orbital (HOMO)/lowest unoccupied molecular orbital (LUMO) energy gaps (**Figure** **1**a), and the side reactions accelerate at high temperatures and in working conditions.^[^ ^23^, ^24^, ^25^, ^26^, ^27^ ^]^ As one of the most attractive SSEs, the sulfide‐based SSEs such as Li_10_GeP_2_S_12_ (LGPS) exhibit high Li^+^ ion conductivity at room temperature, however, seriously suffer from the side reactions with Li metal anode.^[^ ^23^, ^24^, ^25^, ^26^ ^]^ The reduction potential of LGPS is ≈1.7 V versus Li/Li^+^, which is even higher than the general reduction potential of carbonate electrolytes (≈0.7–0.8 V vs Li/Li^+^),^[^ ^24^ ^]^ indicating that unavoidable side reactions would occur while LGPS and Li metal are in contact. These side reactions generally convert the Li/LGPS interface into passivation layer consisted of reduced LGPS products, leading to the formation of Li^+^ and e^−^ mixed conductor phases that undergo Li dendrite growth and decomposition of LGPS.^[^ ^24^, ^26^ ^]^ The growth of Li dendrite could pierce the sulfide‐based SSEs through the pre‐existing defects such as voids, cracks, and grain boundaries. A recent work pointed out that the high electronic conductivity of SSEs could also promote the Li dendrite growth.^[^ ^28^ ^]^ The fast passivation and lithiation lead to the radical phase conversion of the sulfide‐based SSEs and battery failure.

**Figure 1:**
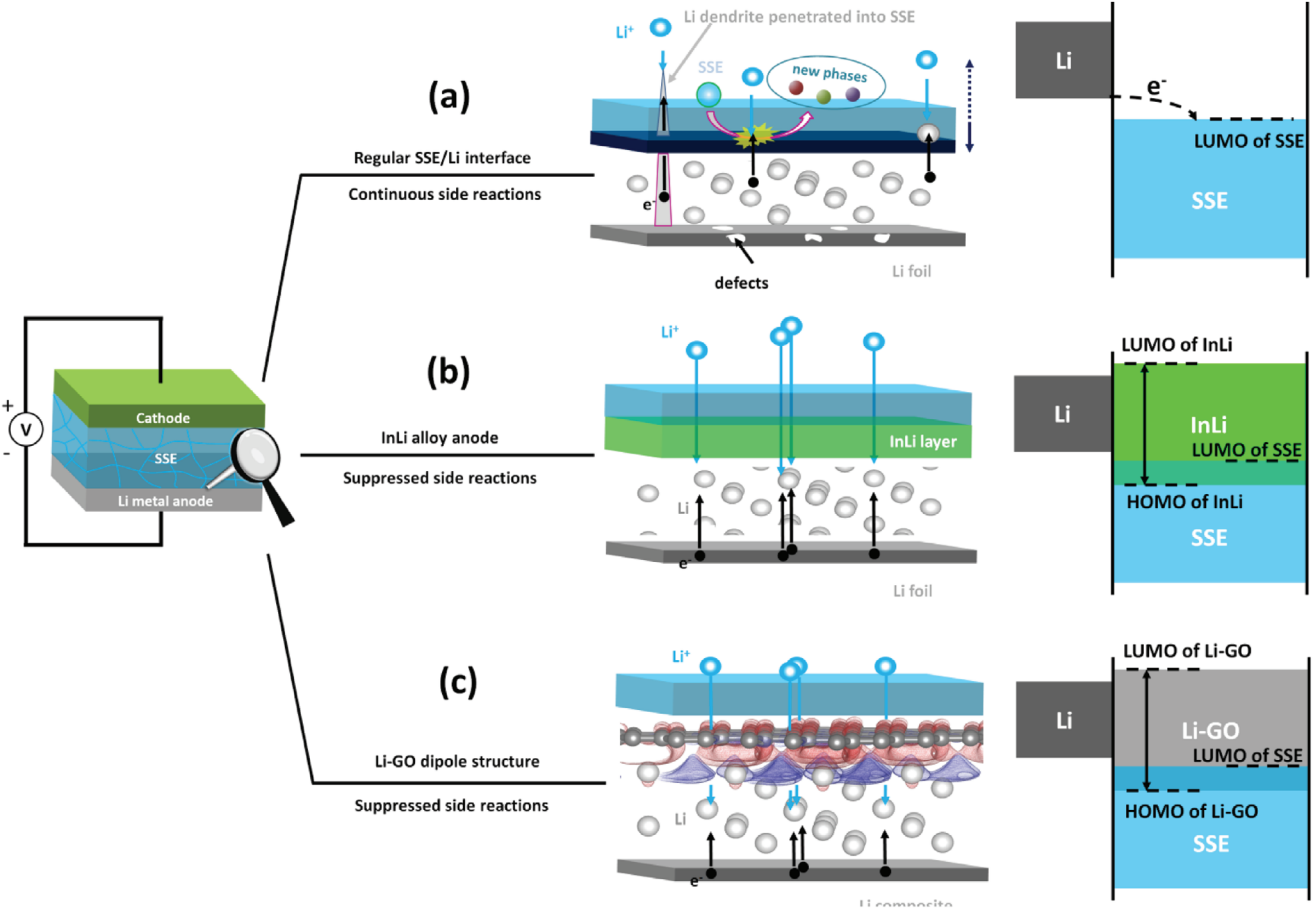
Interfacial issues and energy aspects of the interface between a) Li, b) InLi, or c) Li‐GO dipolar structure and sulfide‐based SSE.

To address the interfacial issues of Li/SSEs, indium (In)‐Li alloy anode with a high anode potential of 0.6 V (vs Li/Li^+^) is generally used to improve the affinity between SSEs and Li and suppress the interfacial passivation of SSEs (Figure 1b).^[^ ^22^ ^]^ However, the use of InLi anode significantly reduces the energy density of SSBs due to the narrowed working voltage window. Employing protective layers such as LiF,^[^ ^29^, ^30^ ^]^ LiI,^[^ ^29^, ^31^ ^]^ and Li_2_HPO_4_^[^ ^32^ ^]^ is another approach to inhibit the side reactions. 3D structures that were widely used in LMBs with liquid electrolytes were also applied to SSBs, leading to the improved interfacial contact between the 3D composite anodes and SSEs.^[^ ^33^, ^34^ ^]^

In this work, we investigated a Li‐GO dipolar structure to enable stable Li/LGPS interface and improve the cycling performances of SSBs. According to the density functional theory (DFT) calculations, the thermodynamically favored Li adsorption around the defects of GO sheets could conduct to the initial deposition of a thin Li layer on the GO sheets, and dipole moments are formed in the as‐obtained Li‐GO structure, due to the charge transfer between the GO and Li layers. The appearance of the interface Li‐GO dipolar structure could significantly improve the stability of Li/electrolyte interface, likely due to an affected HOMO/LUMO energy gap of the Li‐GO structure that stretches over those of Li and electrolyte (Figure 1c). Meanwhile we designed a special functional nanocomposite by incorporating GO sheets into nanosized copper‐cuprous oxide‐GO (Cu‐Cu_2_O‐GO, CCG). The CCG composites were decorated on a 3D Cu net (CCG/Cu), which was used as a functional scaffold to form a composite Li anode by immersing CCG/Cu into molten Li. Using this composite Li@CCG/Cu anode, an SSB using LGPS SSE and LiCoO_2_ (LCO) cathode achieved a long lifespan over 120 cycles with a high coulombic efficiency (CE) > 99.5%.

To study the interaction between Li and GO, an epoxy group was placed at the center of the supercell, and the adsorption energies of Li atom at the sites in the vicinity of the epoxy group were calculated through DFT calculation (**Figure** **2**a). According to the calculated adsorption energies (Table S1, Supporting Information), negative values were only found for the sites close to the epoxy group (sites 1 and 2, Figure 2a), while all the others sites possessed positive values. These results suggest that Li adsorption is thermodynamically favorable around the epoxy group. It should be mentioned that the graphene (G) layer without oxygen functional group is highly lithiophobic, as shown by the Li adsorption energy of 0.517 eV (Table S1, Supporting Information). Similar features were also observed in previous study.^[^ ^35^ ^]^ Thus, the presence of oxygen functional group could provide a locally bonded Li/GO interface, enabling the initial deposition of a thin Li layer on the GO sheet.

**Figure 2:**
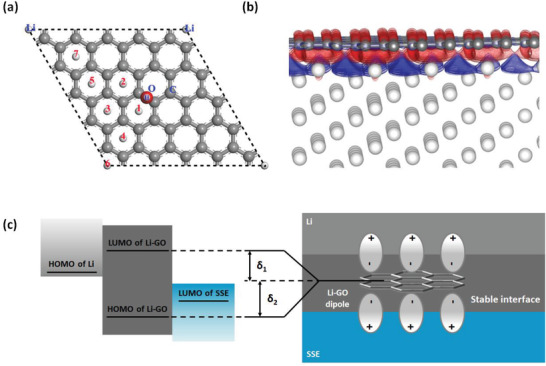
a) Representation of the supercell used for the calculation of Li adsorption on the GO layer. An epoxy group is placed at the center of the supercell. The numbers depict the sites of Li adsorption. b) Calculated charge density *ρ*_diff_ contours between the Li/G interface. The isosurface values of red and blue contours are 0.013 (gain charge) and −0.013 e Å^−3^ (loss charge), respectively. Gray and white spheres represent C and Li atoms, respectively. c) Schematic representation of the possible increase of HOMO/LUMO energy gap of the Li‐GO structure due to the appearance of an interface dipole.

Based on the locally bonded Li/GO interface, further analysis of charge distribution at the interface between Li surface and graphene (G) layer (representing the sites far from the epoxy group) was performed (Figure S1a, Supporting Information). The charge distribution between the G layer and Li surface was studied at an optimal separation distance *d* = 1.85 Å (Figure S1b, Supporting Information). Using this model, we calculated the differential charge density *ρ*_diff_ in the volume comprised between the Li/G interface, which is defined as

(1) $$\begin{array}{c} \rho_{\mathrm{diff}}=\rho_{\mathrm{Li}/C}-\rho_{\mathrm{Li}}-\rho_{C} \end{array}$$

where *ρ*_Li/C_, *ρ*_Li_, and *ρ*_C_ are the charge densities of G/Li intersection zone, separated Li metal surface, and G layer, respectively. The calculated *ρ*_diff_ is shown in Figure 2b, whereas the red and blue contours represent the gain charge (0.013 e Å^−3^) and loss charge (−0.013 e Å^−3^), respectively. A charge transfer from the Li surface toward the G layer is clearly observed, leading to a dipolar structure with positively charged Li surface and negatively charged GO layer. As shown later in this work, this Li‐GO dipolar structure could significantly stabilize the Li/SSE interface, indicating that the appearance of an interface dipole tends to contribute to an increase in the HOMO/LUMO energy gap of the Li‐GO, what stretches over those of Li and sulfide SSE (Figure 2c).

From the intrinsic physics point of view, as shown in Figure 2c, the change of the HOMO and LUMO levels is a direct result of the dipole interactions to the moving charge from the Li metal side to the SSE side, which can be schematically shown in Figure S1c, Supporting Information. The Fermi level of the Li metal is higher than that of the SSE. Without the Li‐GO interface, electrons move directly from the Li metal side to the SSE side without any energy barriers. When the Li‐GO interface is formed, due to the charge redistribution within the interface area, dipoles are formed as previously stated. The dipole moments are in opposite direction at the Li side and the SSE side, which create two energy barriers for the migration of the electrons, thus inhibiting the side reactions at this interface.

The GO sheets were prepared via a modified Hummers method as described in our previous work.^[^ ^4^ ^]^ Transmission electron microscopy photos show that the GO sheets possess a typical crumpled surface with multilayer stacking (Figure S2a, Supporting Information). Functional groups of C—O (286.8 eV) and C=O (287.8 eV) were detected on the C 1s spectrum of X‐ray photoelectron spectrometer (XPS, Figure S2b, Supporting Information). According to the Raman spectrum, the intensity ratio of D band onto G band (*I*_D_/*I*_G_) is 1.05 for the GO sheets used in this work (Figure S2c, Supporting Information). It should be mentioned that the surface functional groups on the GO sheets were not affected by the synthesis of CCG composite. This was verified by the Fourier transform infrared spectra (Figure S2d, Supporting Information).

The CCG composites were decorated on the 3D Cu net to form CCG/Cu through an electrodeposition process (**Figure** **3**a). Scanning electron microscope (SEM) images of the used 3D Cu net are shown in Figure S3, Supporting Information and the process is detailed in the Experimental Section. As comparison, the nanocomposite Cu‐Cu_2_O (CC) without GO was also synthesized on the Cu net to form CC/Cu scaffold. The morphologies of CC/Cu and CCG/Cu are shown in Figure 3b and Figure S4, Supporting Information. The CC composite is mainly consisted of granular substructure (Figure S4a,b, Supporting Information). Meanwhile the GO sheets were uniformly co‐deposited in CCG (Figure 3b and Figure S4c,d, Supporting Information). The presence of Cu_2_O in CC or CCG was clearly identified through X‐ray diffraction (XRD) and XPS. The XRD patterns of CC and CCG composites (powders scrapped‐off from the Cu net substrates) illustrate that metallic Cu metal is the dominant phase, and the signal of Cu_2_O is also present (Figure S5a, Supporting Information). The Auger Cu LMM spectra from XPS further confirmed the ionic state of Cu^+^ in CC and CCG (Figure 3c). Compared to the peak of metallic Cu at ≈568.3 eV, intensive peak of Cu^+^ located at ≈570.0 eV was observed for both CC and CCG,^[^ ^36^ ^]^ confirming the presence of Cu_2_O. Furthermore, a 30 min Ar^+^ etching was applied on the CC and CCG composites, and the obtained Auger Cu LMM spectra are shown in Figure S5b, Supporting Information. The significantly decreased Cu^+^ signals indicate that a core–shell structure with Cu core and Cu_2_O shell was obtained for the microstructures of CC and CCG. The energy‐dispersive X‐ray spectroscopy (EDS) mapping of Cu and O elements gives an overall view on the nanostructure consisted of Cu backbone with Cu_2_O decoration (Figure 3d). It should be mentioned that both the CC and CCG composites were obtained via the electro‐deposition method with an optimal synthesis condition (2 V, 5 min). Other CC composites obtained via the same method with different synthesis conditions were also compared, showing different morphology with similar constituents, i.e., the co‐existence of Cu and Cu_2_O (Figure S6a,b, Supporting Information), and the best Li cycling stability belongs to that on the CC/Cu electrode obtained with the optimal synthesis condition (2 V, 5 min, Figure S6c, Supporting Information).

**Figure 3:**
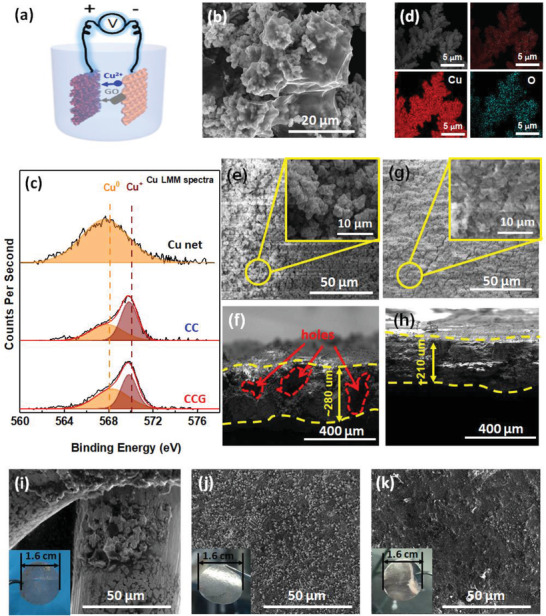
a) Schematic representation of the set‐up for CCG/Cu synthesis. b) SEM image of the CCG composite. c) Auger Cu LMM spectra of Cu net, CC, and CCG. d) EDS mapping of the CC composite. e–h) Top and cross‐sectional SEM images of e,f) CC/Cu and g,h) CCG/Cu after 4 mAh cm^−2^ Li deposition. i–k) SEM images with corresponding digital photos of i) Cu net, j) CC/Cu, and k) CCG/Cu after immersion in molten Li.

The Cu_2_O decoration endows the CC/Cu or CCG/Cu scaffold with lithiophilicity, not only enabling the fast Li infusion in molten Li to form Li composite electrode, but also improving the Li deposition morphology for stabilized cycling. The Li deposition morphology was assessed in liquid carbonate electrolyte. Uneven nucleation and dendrites were clearly observed on the Cu net after 0.25, 1, and 4 mAh cm^−2^ Li deposition (Figure S7a–c, Supporting Information). The sectional view of 4 mAh cm^−2^ Li plating on Cu net further illustrates a precipitated Li deposition on the top surface, leaving large unoccupied void space and highly heterogenous structure (Figure S7d, Supporting Information). These observations indicate that using 3D host without modifying the plating kinetic still engenders uncontrolled volume expansion of Li metal anode. In contrast, owing to the lithiophilicity of Cu_2_O sites, uniform Li deposition was obtained at 0.25 and 1 mAh cm^−2^ on CC/Cu (Figure S7e,f, Supporting Information) and CCG/Cu (Figure S7g,h, Supporting Information). It should be mentioned that the deposited Li layer could be observed on the incorporated GO sheets, leading to the formation of Li‐GO dipole structure (Figure S7g, Supporting Information). At 4 mAh cm^−2^, uniform Li deposition could still be achieved on CC/Cu (Figure 3e) and CCG/Cu (Figure 3g). The cross‐sectional SEM images show that a more compact Li deposition could be achieved in the CCG/Cu scaffold (Figure 3h), compared to that in CC/Cu (Figure 3f). This benefit probably originated from an improved Li adsorption by the GO sheets incorporated in the CCG composite. Based on the compact Li deposition and the reduced Li reactivity by the Li‐GO dipolar structure, much improved Li cycling stability was achieved in CCG/Cu even in the liquid carbonate electrolyte, compared to those achieved by Cu net and CC/Cu (Figures S8 and S9, Supporting Information).

The presence of Cu_2_O in CC/Cu or CCG/Cu is critical to enable fast formation of composite Li electrode through the immersion in molten Li. As shown in Figure 3i, immersing Cu net in molten Li at a high temperature of 350 °C is still inefficient to achieve Li coating on Cu skeleton. In sharp contrast, fast Li infusion into CC/Cu or CCG/Cu could be achieved in less than 20 s at 250 °C, forming a silvery‐white composite Li tablet (inset digital photos in Figure 3j,k). These composite Li electrodes were denoted as Li@CC/Cu and Li@CCG/Cu, respectively. The cross‐sectional SEM images of Li@CC/Cu and Li@CCG/Cu are shown in Figure S10, Supporting Information whereas a similar thickness of ≈140 µm was obtained, corresponding to a Li loading of ≈22.4 mAh cm^−2^. The detail of Li loading calculation is provided in the Supporting Information. The surface components of Li@CC/Cu and Li@CCG/Cu were further analyzed by XRD and XPS. The XRD patterns indicate the consistent presence of metallic Li in Li@CC/Cu and Li@CCG/Cu (Figure S11a, Supporting Information), whereas weak signals of Li_2_O due to the reduction of Cu_2_O by molten Li were also observed. For Li@CCG/Cu, the reduced peaks of C=O (286.8 eV) and C=O (287.8 eV) compared to those of CCG/Cu in C 1s XPS spectra illustrates the reduction of GO sheets by Li (Figure S11b, Supporting Information). In the next section, the as‐obtained composite electrodes were used as anodes in SSBs using sulfide electrolyte to demonstrate the stabilized anode/electrolyte interface via the GO‐incorporated structure.

Several SSBs were assembled using bare Li, Li@CC/Cu, or Li@CCG/Cu as anode, LCO as cathode, and LGPS as electrolyte (Figure S12, Supporting Information). In particular, the anode of InLi alloy was also taken into comparison. Electrochemical impedance spectra (EIS) were carried out to assess the interfacial stability of the investigated anode/SSE interfaces (**Figure** **4**a). Before cycling, similar curves were observed, with a resistance at the high‐frequency region (≈1 × 10^6^ Hz) and a straight line in the rest frequency region (1 × 10^6^ to 1 × 10^−1 ^Hz). These EIS shapes typically depict the state of SSBs before electrochemical activation, whereas the resistance in the high‐frequency region represents the resistance of sulfide SSE (*R*_SE_), and the inactivated electrode/electrolyte interfaces simply display as an infinite charge transfer barrier as shown by the straight line. Upon cycling, the EIS plots significantly changed their shapes, with the appearance of semicircles in the middle‐frequency region (1 × 10^6^ to 1 Hz), which is related to the impedance of existing interfaces (*R*_IN_) in the SSBs. Assuming that the impedance of LCO/electrolyte interface is similar for the investigated samples, thus the overall value of *R*_IN_ could be used to qualitatively compare the polarization of anode/electrode interface. The values of *R*_SE_, *R*_IN_, and *R*_TOTAL_ obtained by fitting the EIS curves using equivalent circuit are shown in Table S2, Supporting Information. After five cycles, a very large *R*_TOTAL_ (16550.0 Ω) was observed for LCO|LGPS|Li, not only caused by the drastic increase of *R*_SE_ due to the deteriorated bulk phase of LGPS, but also affected by the large *R*_IN_ due to the highly accumulated side products. These results clearly show the unstable Li/LGPS interface that limits the application of bare Li anode in SSBs. It is worth noting that the values of *R*_SE_ and *R*_IN_ for LCO|LGPS|Li@CC/Cu (1670.3 and 8319.5 Ω) are much higher than that of LCO|LGPS|Li@CCG/Cu (118.5 and 2292.4 Ω), which demonstrate the critical role of GO to alleviate the side reactions at the anode/LGPS interface. Besides, similar values of *R*_SE_, *R*_IN_, and *R*_TOTAL_ are observed between LCO|LGPS|Li@CCG/Cu and LCO|LGPS|InLi for the cycling up to 50 cycles, showing the stabilized anode/electrolyte interfaces for these two anodes.

**Figure 4:**
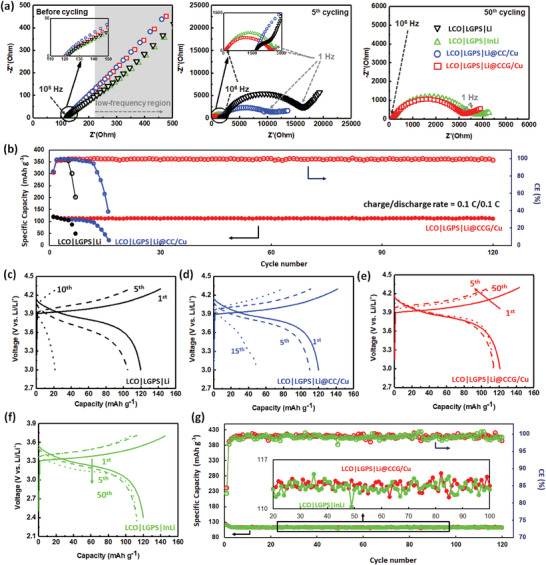
a) Nyquist plots of LCO|LGPS|Li, LCO|LGPS|Li@CC/Cu, LCO|LGPS|Li@CCG/Cu, and LCO|LGPS|InLi upon cycling. b) Cycling performances of LCO|LGPS|Li, LCO|LGPS|Li@CC/Cu, and LCO|LGPS|Li@CCG/Cu. c–f) Charge–discharge curves of c) LCO|LGPS|Li, d) LCO|LGPS|Li@CC/Cu, e) LCO|LGPS|Li@CCG/Cu, and f) LCO|LGPS|InLi at different cycles. g) Cycling performances of LCO|LGPS|Li@CCG/Cu and LCO|LGPS|InLi.

The increase of the HOMO/LUMO energy gap of the Li‐GO structure is observed by the open@circuit voltage (OCVs) of the SSBs. As shown in Figure S13, Supporting Information, the OCV of the SSB using Li@CC/Cu is lower than that using bare Li, due to a more continuous potential evolution at the interface between Li@CC/Cu and sulfite SSE. Meanwhile the OCV for the cell using Li@CCG/Cu is raised above that with bare Li, demonstrating the increased HOMO/LUMO energy gap by the presence of Li‐GO structure. This increased energy gap leads to stabilized anode/electrolyte interface without affecting the charge transfer kinetic, as shown by the improved cycling performances of the SSB using Li@CCG/Cu. The cycling performances of the investigated SSBs are shown in Figure 4b. Fast capacity and CE fading were observed for LCO|LGPS|Li and LCO|LGPS|Li@CC/Cu. The charge–discharge curves of LCO|LGPS|Li (Figure 4c) and LCO|LGPS|Li@CC/Cu (Figure 4d) consistently depict the drastically increased polarization causing the impedance failure of these cells, clearly indicating the unstable interface of Li or Li@CC/Cu with LGPS. It should be mentioned that the cycling stability of Li anode on CC/Cu was comparable to that on CCG/Cu in liquid carbonate electrolyte (Figure S8, Supporting Information). Thus, the poor cycling stability of SSB using Li@CC/Cu indicates that the side reactions between Li and LGPS are probably more serious than in liquid electrolyte. In sharp contrast, stable cycling was achieved for LCO|LGPS|Li@CCG/Cu, exhibiting a discharge capacity of 112.4 mAh g^−1^ at the 120th cycle (corresponding to a capacity retention of 92.9%) with a high average CE > 99.5%. Though stable cycling was also achieved for LCO|LGPS|InLi (Figure 4g), its energy density is lowered due to the narrowed voltage window with the anode redox potential of In/In^3+^ (0.6 V vs Li/Li^+^, Figure 4f). These results demonstrate the distinct advantage of the GO‐incorporated structure to protect Li metal anode in keeping the high energy density of SSBs.

The stabilized anode/electrolyte interface in LCO|LGPS|Li@CCG/Cu not only inhibited the side reactions causing high interfacial impedance, but also maintained the structure of LGPS. The latter was verified by postmortem XPS and SEM analyses for the SSBs after ten cycles. The LGPS electrolytes dissembled from the cycled LCO|LGPS|Li, LCO|LGPS|Li@CC/Cu, and LCO|LGPS|Li@CCG/Cu were denoted as LGPS_Li_, LGPS_CC_, and LGPS_CCG_, respectively. Compared to the pristine LGPS (**Figure** **5**a), the LGPS_Li_ shows two reduced germanium species (≈28.6 and ≈27.0 eV) in Ge 3d spectra (Figure 5b). The two additional peaks may be ascribed to the formation of Ge*^x^* ^+^ (*x* = 0, 1, 2, or 3), which is difficult to be differentiated due to the overlapping.^[^ ^25^ ^]^ Meanwhile, reduced phosphorus (≈130.5 eV) and Li_3_P (≈126.0 eV) were clearly observed besides the major peak of PS_4_‐tetrahedra (≈132 eV) on the P 2p spectrum of LGPS_Li_.^[^ ^26^, ^37^ ^]^ In addition, the peaks of Li_2_S (≈160 eV) and metallic Li (≈54.2 eV) were observed on the S 2p and Li 1s spectra of LGPS_Li_, respectively. All of these reduction products from LGPS_Li_ clearly indicate the serious side reactions of LGPS with Li metal anode. Similar products were also observed for LGPS_CC_ (Figure 5c). Distinctly, the surface components of LGPS_CCG_ are highly similar to those of pristine LGPS (Figure 5d). According to the calculations of the unreduced peak areas onto the total peak areas, the values of Ge, P, S, and Li elements for LGPS_CCG_ are highly close to those of LGPS (100%), significantly outperforming those of LGPS_Li_ and LGPS_CC_ (Figure 5e). These results demonstrate the significantly suppressed side reactions at the anode/electrolyte interface by the GO‐incorporated structure. Consistently, intact surface morphology was kept for LGPS_CCG_ (Figure 5i), compared to that of pristine LGPS (Figure 5f). In contrast, tremendous whisker Li and cracked flakes were formed on LGPS_Li_ (Figure 5g) and LGPS_CC_ (Figure 5h). Though the Li cycling stability could be enhanced on the CC/Cu structure in liquid carbonate electrolyte (Figure S8, Supporting Information), the failed LGPS_CC_ surface indicates that reducing the reactivity of Li metal anode via the Li‐GO dipolar structure is indispensable for the SSBs using LGPS as electrolyte. Also, the poor interfacial contact at the Li/LGPS interface is another issue that critically affects the cycling stability of Li metal anode in SSBs. As shown in Figure S14, Supporting Information, clear interfacial disintegration with inner holes was observed at the cycled Li/LGPS interface, while a tight interfacial contact was maintained for the cell using Li@CCG/Cu anode. The latter was due to the Li‐GO dipole structure that keeps the deposited Li from the contact with LGPS, suppressing continuous side reactions that deteriorate the Li/LGPS interface.

**Figure 5:**
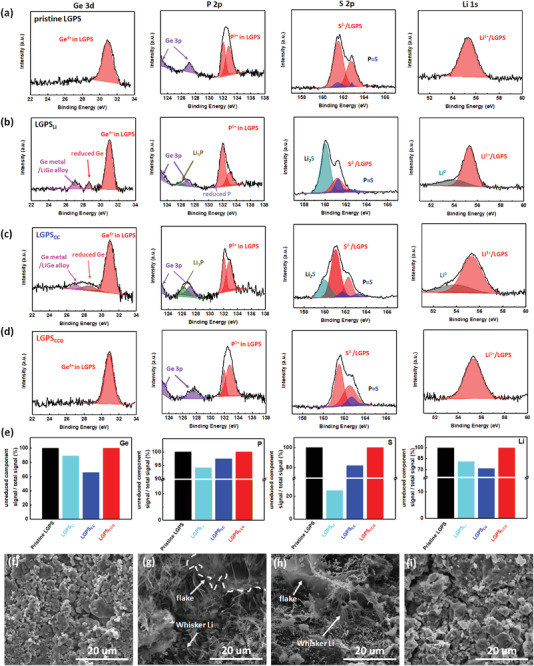
a–d) Ge 3d, P 2p, S 2p, and Li 1s XPS spectra of a) pristine LGPS, b) LGPS_Li_, c) LGPS_CC_, and d) LGPS_CCG_. e) Ratio of the unreduced component peak areas onto the total peak areas of the investigated samples for Ge, P, S, and Li XPS spectra. f–i) SEM images of f) pristine LGPS, g) LGPS_Li_, h) LGSP_CC_, and i) LGPS_CCG_.

In summary, we investigated the possibility of stabilizing the Li/SSE interface via the Li‐GO dipolar structure. We demonstrated via DFT calculation that, after the initial Li deposition around the defects of GO sheets, the Li‐GO dipolar structure could be achieved due to the electron‐withdrawing ability of GO acting on Li, significantly lowering the reactivity of the Li/SSE interface. Based on this benefit, the GO‐incorporated lithiophilic host structure, CCG/Cu, was synthesized and used to form the Li@CCG/Cu anode. This composite anode showed prominent effect to stabilize the anode/electrolyte interface in SSBs using LGPS as electrolyte and LCO as cathode, exhibiting a stable cycling with a high average CE > 99.5% over 120 cycles. This work described a possible remedy to improve the cycling stability of high‐energy‐density SSBs using Li metal as anode, bypassing the expensive In protective layer that reduces the energy density of SSBs.

## Conflict of Interest

The authors declare no conflict of interest.

## Supporting information

Supporting Information
