# Supplementary material for: Improving the Interfacial Stability between Lithium and Solid‐State Electrolyte via Dipole‐Structured Lithium Layer Deposited on Graphene Oxide
Source: Adv Sci (Weinh). 2020 May 18;7(13):2000237. doi: 10.1002/advs.202000237 (PMC7341093; doi:10.1002/advs.202000237)
Supplement: Supplementary file 1 — Supporting Information [file ADVS-7-2000237-s001.pdf]

Copyright WILEY-VCH Verlag GmbH & Co. KGaA, 69469 Weinheim, Germany, 2020.

Supporting Information

Improving the interfacial stability between Lithium and Solid State Electrolyte via Dipole-Structured Lithium layer deposited on Graphene Oxide

Muqin Wang, Zhe Peng, Wenwei Luo, Qiang Zhang, Zhendong Li, Yun Zhu, Huan Lin, Liangting Cai, Xiayin Yao, Chuying Ouyang,* Deyu Wang**

M. Wang, Z. Peng, Q. Zhang, Z. Li, Y, Zhu, H. Lin, L. Cai, X. Yao, D. Wang
Ningbo Institute of Materials Technology and Engineering, Chinese Academy of Sciences, Ningbo 315201, China.
E-mail: pengzhe@nimte.ac.cn; wangdy@nimte.ac.cn

W. Luo, C. Ouyang
Department of Physics, Laboratory of Computational Materials Physics, Jiangxi Normal University, Nanchang 330022, China.
E-mail: cyouyang@jxnu.edu.cn

Experimental Section

Material preparations:

Pristine Cu net of 150 meshes was cut into an area of 3 × 3 cm^2 and cleaned in deionized water and ethanol consecutively via ultrasonic for 15 minutes. To form the CC structure, two cleaned Cu nets were separately used as working and counter electrodes in an aqueous bath of 0.15 M $CuSO_4•5H_2O$. Potentiostatic deposition was then carried out under a voltage of 2 V for 5 min in such a two-electrode system. The structure of CCG has been synthesized in the same electrodeposition system, by adding a homemade graphene oxide (GO) in the aqueous solution with a concentration of 0.5 g L^{-1}. The fabrication process of the homemade GO could be found in our previous work.[1] After the deposition process, the samples were washed by deionized water and ethanol.

Electrochemical measurements:

For SSBs, the synthesis of LGPS powders can be found in previous works.[2,3] Single-layer pellets containing LGPS (100 mg) were obtained by pressing under 240 MPa to form the electrolytes. To obtain the cathode, LGPS and LCO powders were manually mixed at a weight ratio of 30:70, and then homogeneously spread on the top side of the single-layer LGPS pellet. The capacity loading of the as-obtained cathode is ~ 0.33 mAh cm^{-2} (120 mAh g^{-1}) of active LCO materials. Li foil, Li@CC/Cu, Li@CCG/Cu or InLi anode was attached to the bottom side of the single-layer LGPS pellet. The SSB was finally obtained by pressing the cathode (top), LGPS pellet (middle) and anode (bottom) under 360 MPa. The InLi anode was in-situ formed during the pressing process by placing an In layer between the Li foil and LGPS pellet. All the pressing processes were carried out by placing the SSB components in a polyteteafluoroethylene mold (diameter 10 mm) between two stainless steel current collectors, in a dry argon-filled glove box with H_2O and O_2 contents < 0.1 ppm. For the SSBs using Li foil, Li@CC/Cu or Li@CCG/Cu as anode, the cells were cycled between 3.0 and 4.3 V vs. Li/Li^+ with charge/discharge rates of 0.1C/0.1C. For the SSBs using InLi as anode, the cells were cycled between 2.4 and 3.7 V vs. Li/Li^+ with charge/discharge rates of 0.1C/0.1C. All the tests of SSBs were performed in a sealed system under Ar atmosphere, using a battery testing system (LandCT2001 from LAND electronics Co, Ltd).

For the cycling of Li metal in liquid carbonate electrolyte, 50 μL electrolyte (1 M $LiPF_6$ in 1:1 ethylene carbonate, EC and diethyl carbonate, DEC, with vol. 5% Fluoro-ethylene carbonate, FEC, Sigma-Aldrich Co, LLC) was deposed in Celgard separator film (diameter: 18 mm; thickness: 20 μm). Coin cells CR2032 were used for cell assembly. The $LiFePO_4$ (LFP) electrodes used in liquid electrolyte tests were obtained by mixing $LiFePO_4$:Super-P:PVDF (8:1:1) in NMP. The slurries were cast on aluminum foils, followed by vacuum drying at 120 ℃ for 12 h to obtain the electrodes. The capacity

loading of the obtained LFP cathode is ~ 0.85 mAh cm^{-2} (170 mAh g^{-1}). The cells were cycled between 2.5 and 4.4 V vs. Li/Li^+ with charge/discharge rates of 0.5C/0.5C.

The EIS spectra were measured using a potentiostat/galvanostat 1470E equipped with a frequency response analyzer 1455A from Solartron. The EIS measurements were performed in the frequency range from 1×10^6 to 1×10^{-1} Hz with a voltage perturbation of 15 mV. The equivalent circuit was simulated by the Zview software.

Electrode characterizations:

The crystalline phase of the prepared samples was characterized by XRD with a Bruker D8 advanced diffractometer using Cu Kα (λ = 1.5406 Å) radiation (Bruker axs, D8 Advance). Surface analysis was conducted with a PHI 3056 XPS, which was excited by a Mg Kα radiation source at a constant power of 100 W (15 kV and 6.67 mA). The microstructure analysis was performed using SEM (FEI, QUANTA 250 FEG) and TEM (FEI, Tecnai F20, 200 kV). Raman spectra were obtained with a Renishaw inVia reflex Raman spectrometer at an excitation wavelength of 532 nm from an Nd:YAG (neodymium-doped yttrium aluminum garnet) laser operating at 100 mW. FTIR spectra from 400 to 4000 cm^{-1} were collected on a Nicolet 6700 spectrometer (Thermo Fisher Scientific) in transmission model when the powders of the CC or CCG were pressed to pellets with KBr.

Density functional theory calculations:

DFT calculations were performed using the Vienna ab initio simulation package (VASP) code.[4] The projector augmented wave (PAW) pseudopotentials[5] were used to describe the atomic potential while the electron exchange-correlation was described in the generalized gradient approximation (GGA) using the Perdew-Burke-Ernzerhof (PBE).[6] The energy cutoff for the plane wave expansion was 520

eV. The lattice parameters and the atomic positions were fully optimized until the forces are converged to 0.01 eV Å^{-1}. The Li(111) surface was modeled with a slab model that contains 11 layers of Li atoms and separated with a vacuum layer of about 15 Å. The lattice mismatch of 4×4 graphene layer and 2×2 Li(111) is only about 4.2%, and thus the interface was modeled with them. The Monkhorst-Pack[7] k-point sampling method was used for the integration in the irreducible Brillouin zone and the density of the k-mesh was thicker than 0.03 Å^{-1} for all cases. The van der Waals (vdW) correction was included in the calculation using the DFT-D3 of Grimme.[8]

Table S1. The calculated adsorption energies (in eV) for Li adsorption at different sites of the GO surface. These sites are differentiated by their distance (in Å) to a fixed epoxy group.

Adsorption sites	Distance (Å)	E_{ad} (eV)
1	1.233	-0.475
2	2.136	-1.140
3	3.263	0.096
4	3.700	0.160
5	4.447	0.219
6	6.617	0.227
7	6.409	0.255
pristine graphene	∞	0.517

Table S2. Values of R_{SE}, R_{IN} and R_{TOTAL} for the investigated SSBs, the values were obtained by fitting the EIS data using equivalent circuit.

		**Before cycling**	**5th**	**50th**
R_{SE} (Ω) (interrupt along the *Z'*-axis)	LCO|LGPS|Li	122.3	1707.4	-
	LCO|LGPS|Li@CC/Cu	121.6	1670.3	-
	LCO|LGPS|Li@CCG/Cu	120.6	118.5	123.3
	LCO|LGPS|InLi	120.1	117.6	122.8
R_{IN} (Ω) (semicircles in the	LCO|LGPS|Li	-	14842.6	-
	LCO|LGPS|Li@CC/Cu	-	8319.5	-

middle-frequency region)	LCO|LGPS|Li@CCG/Cu	-	2292.4	3074.6
	LCO|LGPS|InLi	-	2470.0	3474.1
R_{TOTAL}(Ω)	LCO|LGPS|Li	122.3	16550.0	-
	LCO|LGPS|Li@CC/Cu	121.6	9989.8	-
	LCO|LGPS|Li@CCG/Cu	120.6	2410.9	3197.9
	LCO|LGPS|InLi	120.1	2587.6	3596.9

Calculation details of Li loading in Li@CC/Cu or Li@CCG/Cu electrode

By measuring the mass of Li@CC/Cu or Li@CCG/Cu before/after the Li infiltration, the Li loading L (mAh cm^{-2}) could be calculated as follows:

$$L = \frac{\Delta m \times F}{M \times A \times 3.6}\ (\mathrm{mAh}\ cm^{-2})$$

where Δ m (g) is the mass of Li@CC/Cu or Li@CCG/Cu before/after the Li infiltration, A (cm^2) is the surface of the electrode, F (C mol^{-1}) is the Faraday constant, M (g mol^{-1}) is the molar mass of Li.

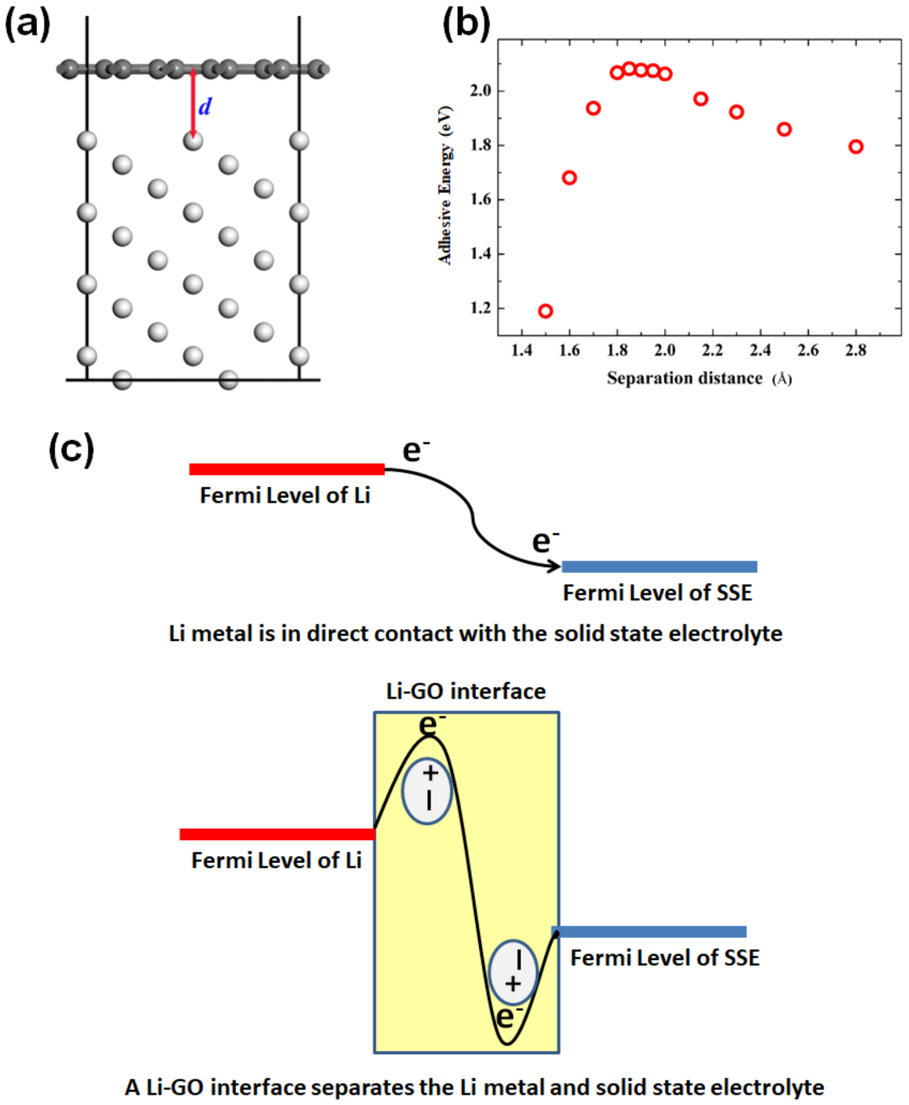

Figure S1. (a) Ball and stick representation of the Li/G interface. The white and gray spheres represent Li and C atoms, respectively. The symbol d represents the distance between the G layer and Li surface; (b) Adhesive energy between the G layer and Li surface as a function of d; (c) Interfacial energy levels of the Li/SSE interfaces with/without the Li-GO dipole structure.

The Li/G interface is simulated by placing a single G layer on the Li(111) facet. The adhesive energy (defined as $E_{adh} = E_C + E_{Li} - E_{Li/C}$) was calculated as a function of the separation distance, d. The highest adhesive energy was found at d = 1.85 Å, which was considered as the optimal separation distance that was used in further DFT calculation.

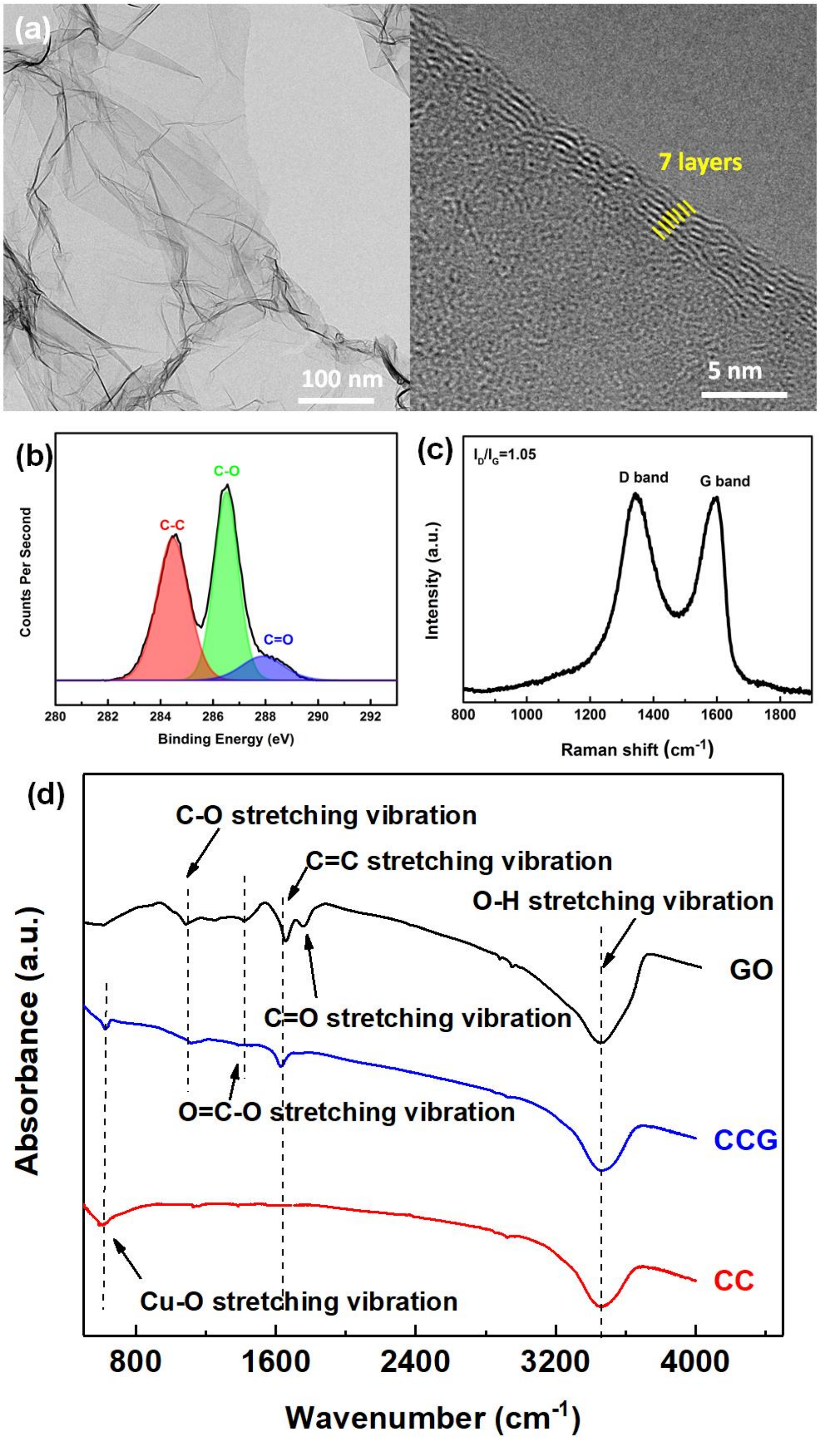

Figure S2. (a) TEM images, (b) C 1s XPS and (c) Raman spectra of the homemade GO sheets; (d) FTIR spectra of GO, CC (nano-composite Cu-Cu_2O without GO) and CCG.

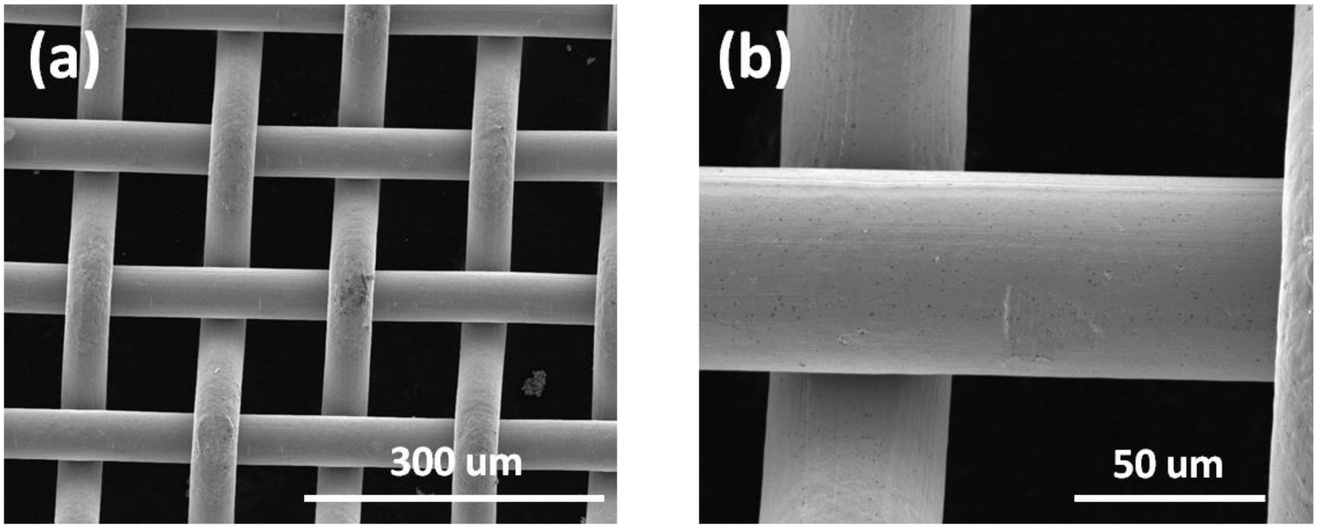

Figure S3. SEM images of a pristine Cu net.

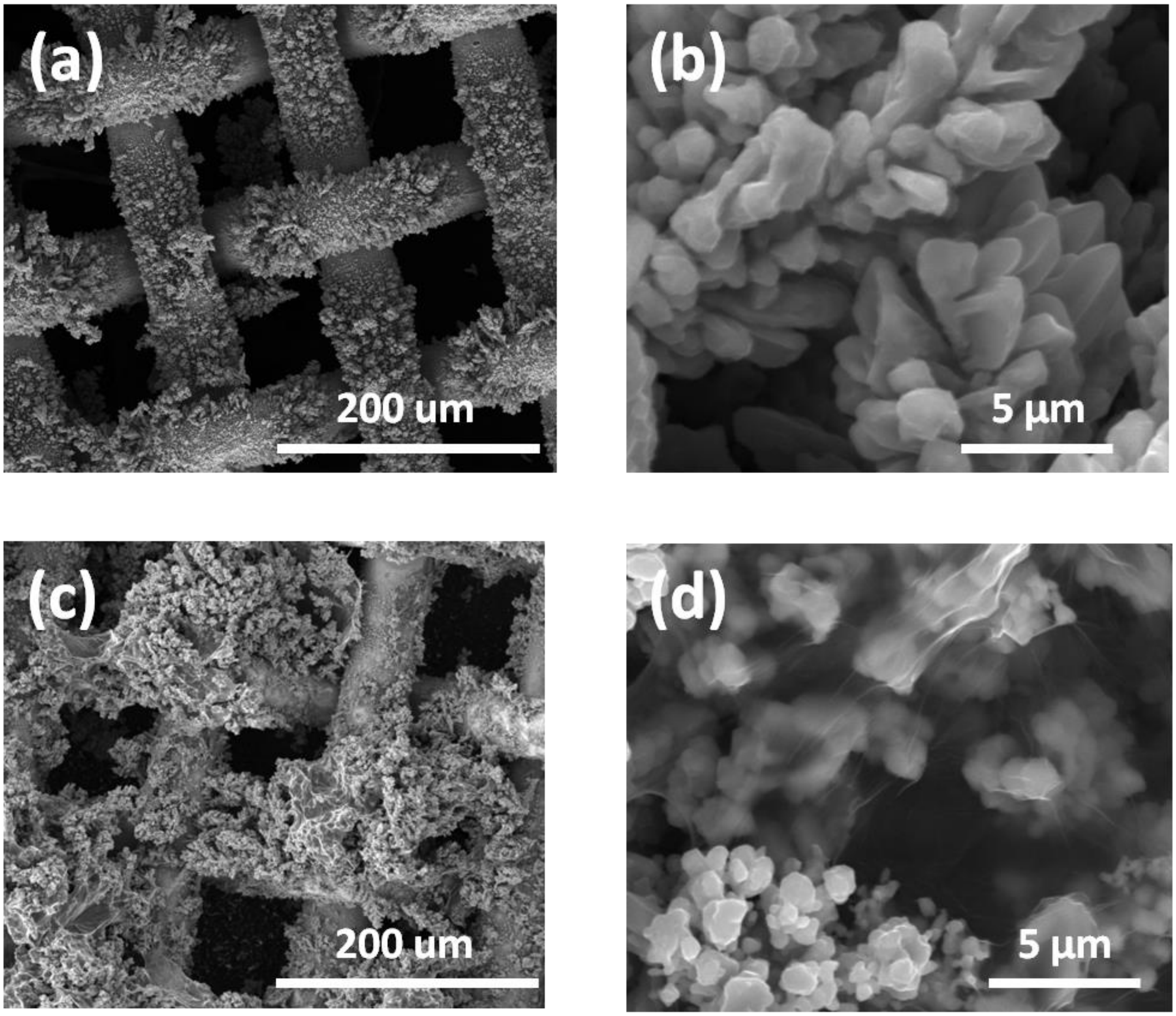

Figure S4. Top-view SEM images of (a, b) CC/Cu and (c, d) CCG/Cu.

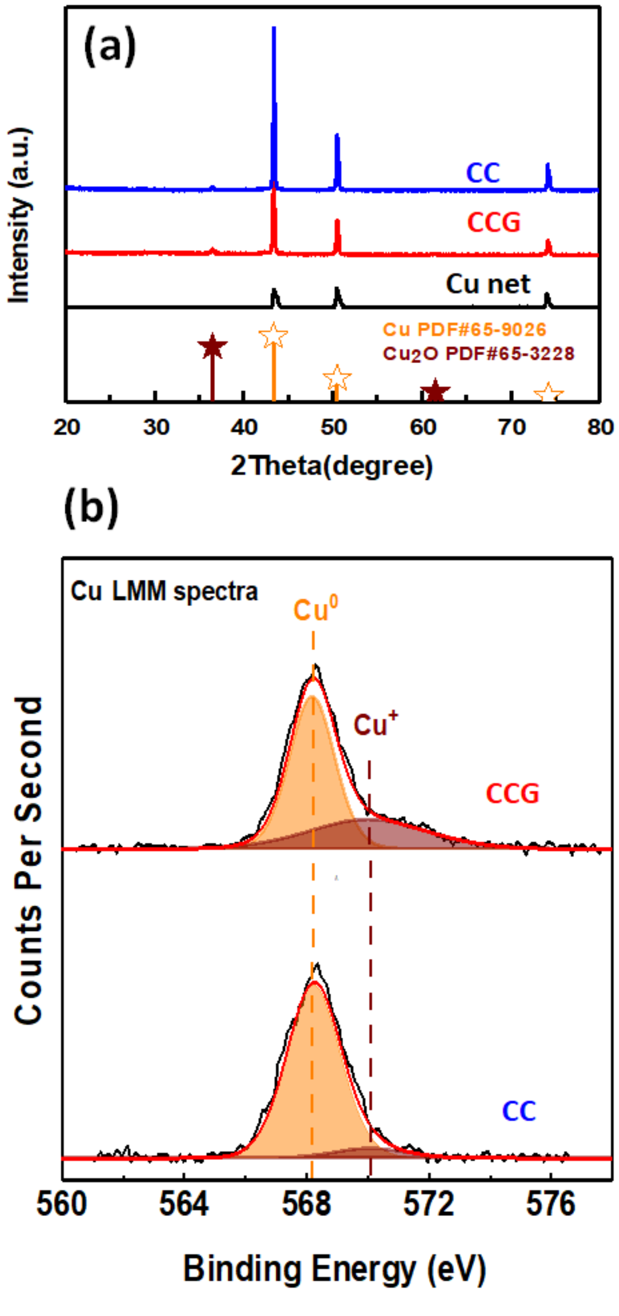

Figure S5. (a) XRD patterns of Cu net, CC and CCG, (b) Auger Cu LMM spectra of CC and CCG exposed to 30 min Ar^+ etching.

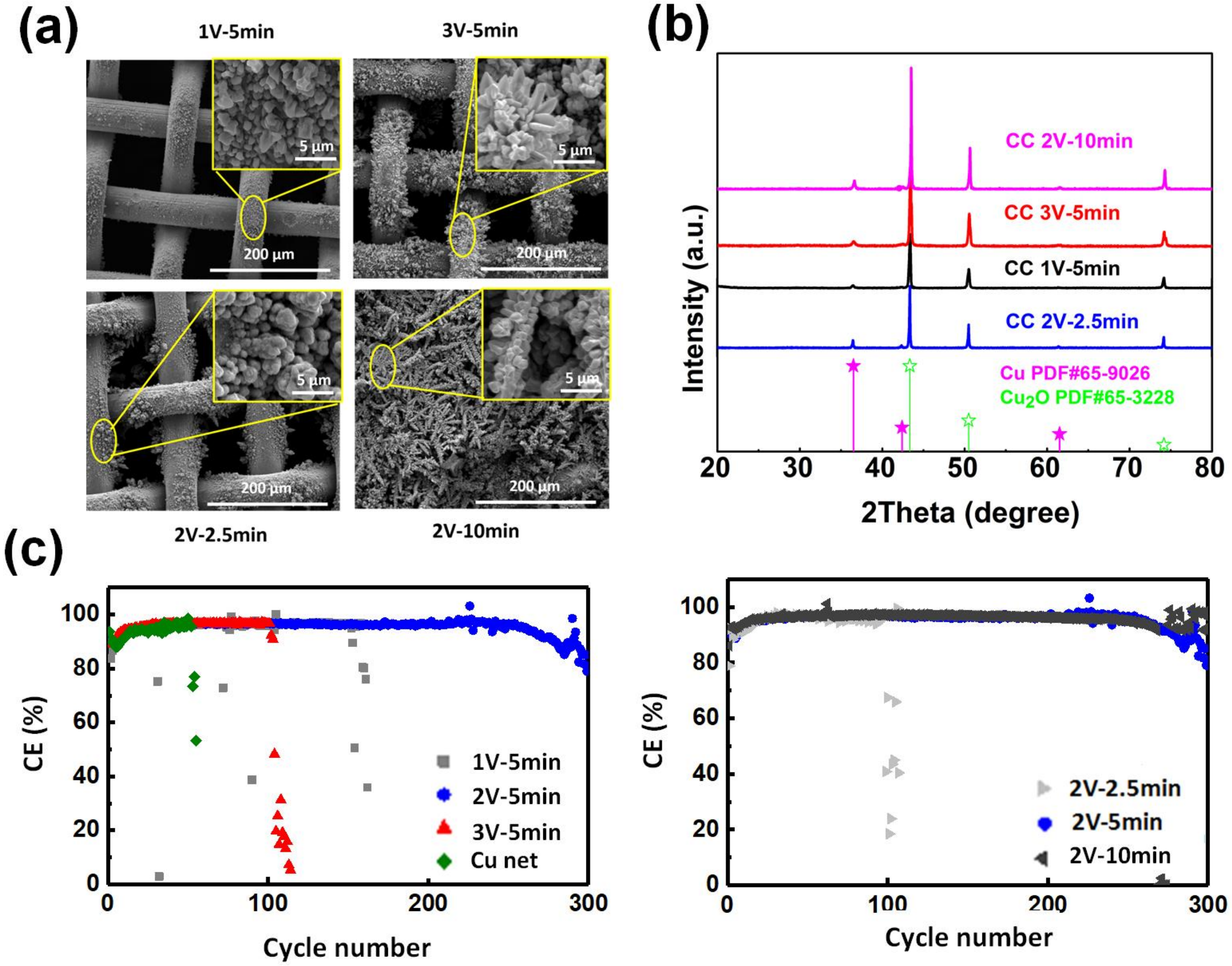

Figure S6. (a) SEM images and (b) XRD patterns of the CC composites obtained via the electro-deposition method with different synthesis conditions; (c) Li cycling stability on the CC/Cu electrodes with these composites. The cycling tests were carried out at 0.5 mA cm^{-2} for a fixed capacity of 1 mAh cm^{-2}.

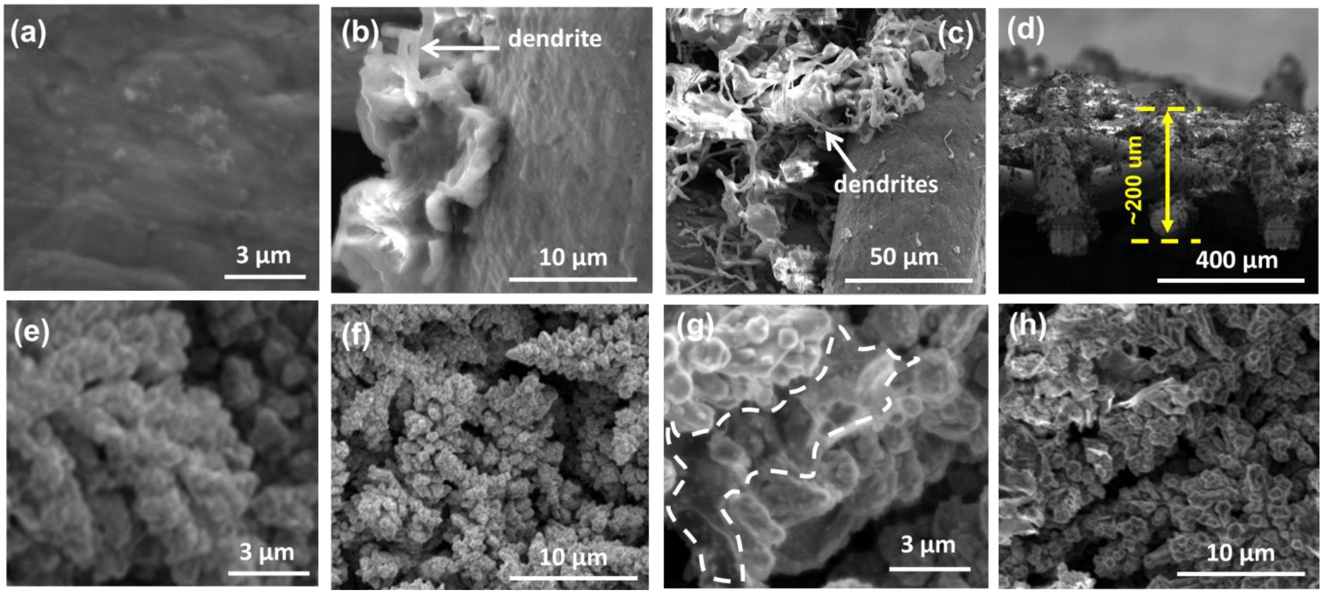

Figure S7. (a-c) SEM images of Li deposits at (a) 0.25, (b) 1 and (c) 4 mAh cm^{-2} on Cu net; (d) Sectional SEM image of Cu net after a Li plating of 4 mAh cm^{-2}; (e, f) SEM images of Li deposits at (e) 0.25 and (f) 1 mAh cm^{-2} on CC/Cu; (g, h) SEM images of Li deposits at (g) 0.25 and (h) 1 mAh cm^{-2} on CCG/Cu. A liquid carbonate electrolyte (1 M $LiPF_6$ in 1:1 EC:DEC with vol. 5% FEC) was used for performing the Li electroplating.

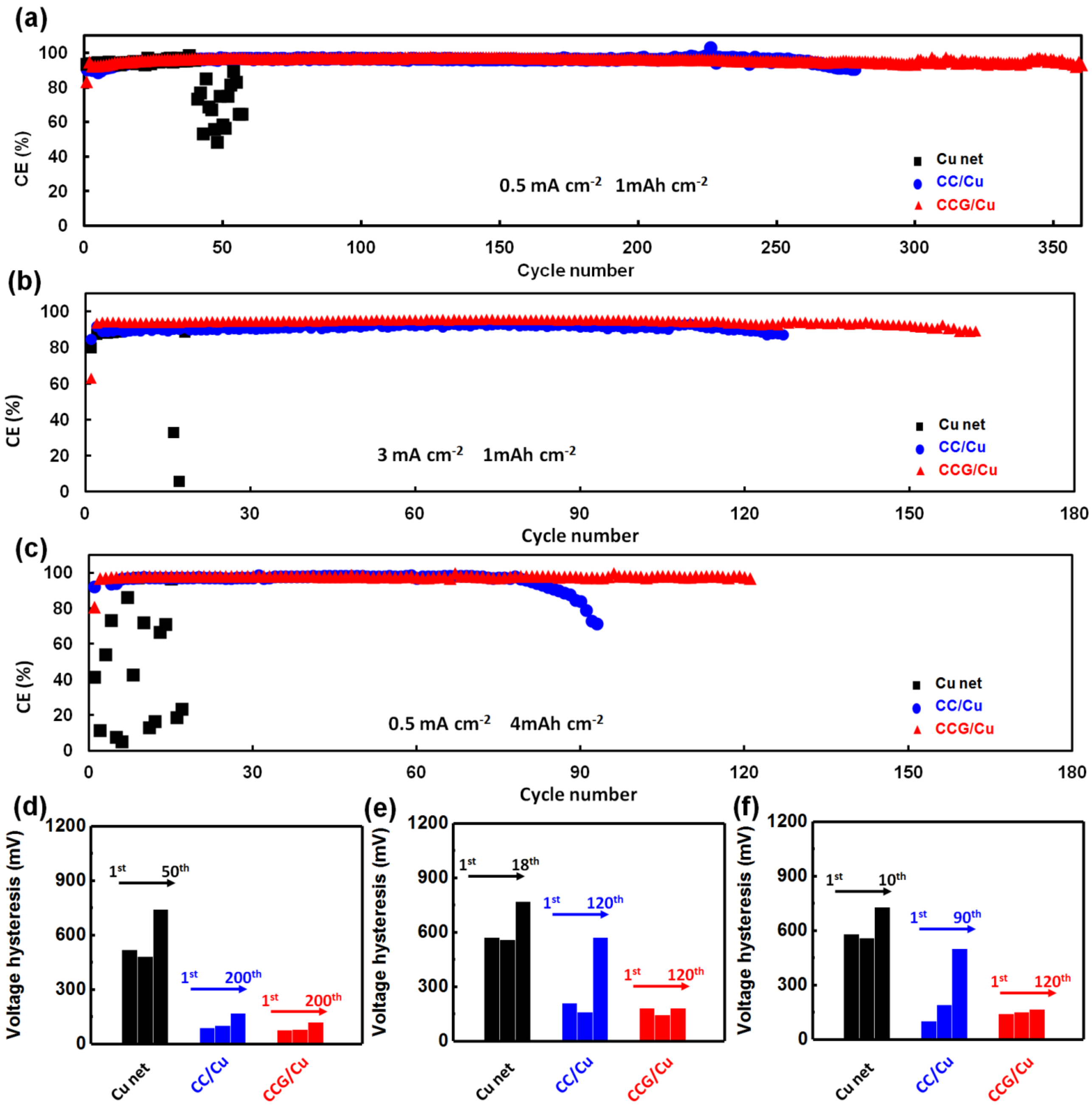

Figure S8. (a-c) CEs of Li plating/stripping on Cu net, CC/Cu and CCG/Cu electrodes at (a) 0.5 mA cm^{-2} - 1 mAh cm^{-2}, (b) 3 mA cm^{-2} - 1 mAh cm^{-2}, and (c) 0.5 mA cm^{-2} - 4 mAh cm^{-2}; (d-f) Voltage hysteresis of Li plating/stripping on Cu net, CC/Cu and CCG/Cu electrodes, at (d) 0.5 mA cm^{-2} - 1 mAh cm^{-2}, (e) 3 mA cm^{-2} - 1 mAh cm^{-2}, and (f) 0.5 mA cm^{-2} - 4 mAh cm^{-2}.

Cycling Li CEs have been assessed using Cu net, CC/Cu and CCG/Cu as working electrodes, on which repeated Li plating/stripping were performed. At 0.5 mA cm^{-2} for a fixed capacity of 1 mAh cm^{-2}, a stable CE of ~ 97.5% was remained over 350 cycles for CCG/Cu, which outperformed that of ~ 96.1% for CC/Cu over a cycling time of 260 cycles (**Figure S8**a, Supporting Information). In sharp contrast, a fast drop of CE was achieved on Cu net for a cycling less than 50 cycles. The poor cycling CE on Cu net is a direct indicator of massive side reactions leading to thick and highly impeding "dead Li" layer. Consistently, a fast increase of voltage hysteresis from ~ 510 mV of the 1st cycle to ~ 740 mV of the 50th cycle was achieved on Cu net (**Figure S8**d, Supporting Information). The voltage hysteresis was much reduced and stabilized for CC/Cu, from ~ 90 mV of the 1st cycle to ~ 170 mV of the 200th cycle. Such an improved stability is based on the alleviated current density and uniform Li plating/stripping morphology, enabled by the high-surface-area Cu nanostructures and lithiophilic Cu_2O sites. Further enhancement was achieved by CCG/Cu, with small voltage hysteresis ranging from ~ 75 mV of the 1st cycle to ~120 mV of the 200th cycle. The latter demonstrates that the incorporation of GO sheets in CCG has a great impact on suppressing side reactions between Li metal and electrolyte.

Rate ability of the investigated electrodes has been assessed at a higher current density of 3 mA cm^{-2} for 1 mAh cm^{-2}. Fast decay of Li CE on Cu net clearly reflects that the raw 3D structure is not able to keep stable Li plating/stripping at high rate cycling (**Figure S8**b, Supporting Information), leading to drastic polarization caused by Li/electrolyte interfacial failure (**Figure S8**e, Supporting Information). For CCG/Cu, the ability of maintaining high CE and low voltage hysteresis was kept at 3 mA cm^{-2}, affording a stable cycle over 160 cycles with a high CE of ~ 95% and a low voltage hysteresis stabilized at ~ 180 mV (**Figures S8**b and **S8**e, Supporting Information). The better performance of CCG/Cu compared to that of CC/Cu also demonstrate the effect of GO for enhancing the CE at high rate cycling.

For achieving high energy density of the composite anode, the ability of accommodating large Li amount inside the host structure is important. The latter has been verified by cycling Li metal at a high capacity of 4 mAh cm^{-2} on the investigated electrolytes (**Figure S8**c, Supporting Information). At such a high Li loading, only parasite CE evolution was achieved for Cu net, indicating the uneven Li storage inside the raw 3D host. This feature has been revealed in **Figure S7**d, Supporting Information, whereas the majority of Li deposition was accumulated at the top surface of Cu net due to its lithiophobic property. Consequently, very large polarization was induced at the initial cycling and upon cycling (**Figure S8**f, Supporting Information). In contrast, CCG/Cu maintained a stable cycle over 120 cycles with an average CE of ~ 97.6% (**Figure S8**c, Supporting Information) and low voltage hysteresis ~ 160 mV (**Figure S8**f, Supporting Information).

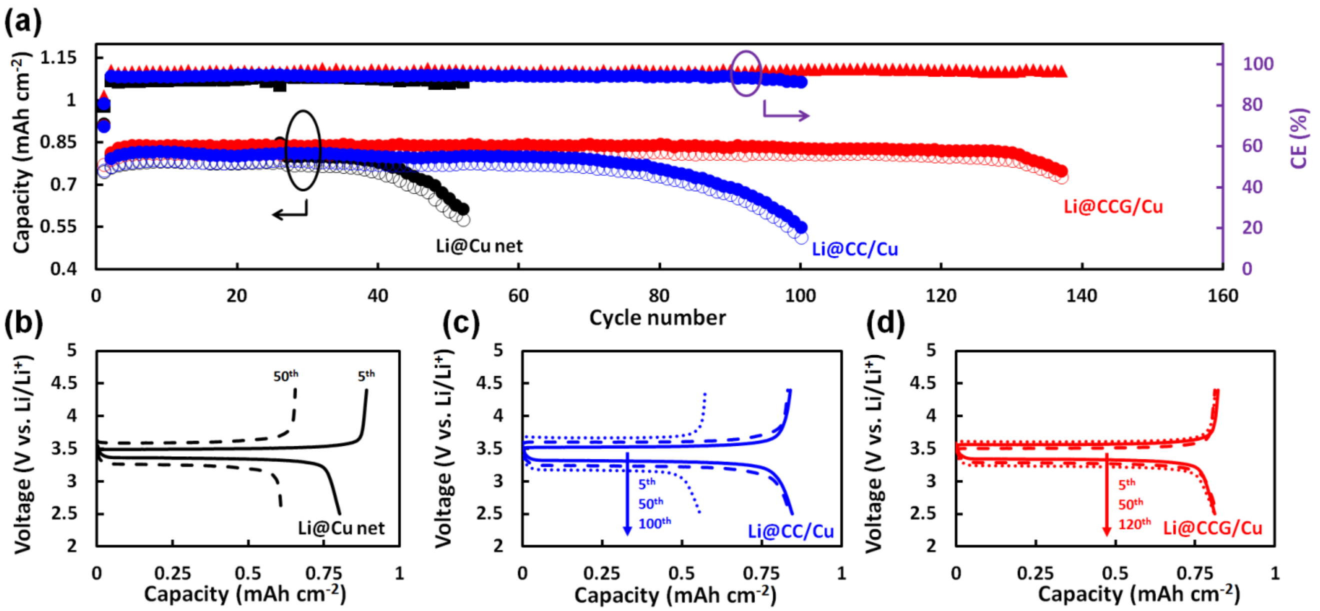

Figure S9. (a) Capacity retentions and CEs of the full cells using $LiFePO_4$ (LFP) as cathode and Li@Cu net, Li@CC/Cu or Li@CCG/Cu as anode; (b-d) Voltage profiles for the cells using (b) Li@Cu net, (c) Li@CC/Cu or (d) Li@CCG/Cu as anode. The Li@Cu net, Li@CC/Cu or Li@CCG/Cu anode

was obtained by plating 2.5 mAh cm^{-2} Li on the corresponding structure. The mass loading of the LFP cathodes was ~ 0.85 mAh cm^{-2}.

Full cells using Li@Cu net, Li@CC/Cu or Li@CCG/Cu as composite anode and $LiFePO_4$ (LFP) as cathode have been assembled. To clearly demonstrate the ability of Li protection, a limited Li amount of 2.5 mAh cm^{-2} was electrochemically pre-plated on Cu net, CC/Cu or CCG/Cu to form the composites anode, and then paired with LFP cathode (mass loading ~ 0.85 mAh cm^{-2}). The cell using Li@Cu net showed a fast fading capacity below 0.6 mAh cm^{-2} at the 50th cycle (**Figure S9**a, Supporting Information), due to the unprotected Li located at the top surface of Cu net, which directly reacted with electrolyte to form highly impeding "dead Li" causing enlarged polarization (**Figure S9**b, Supporting Information). Improved performance was achieved by the cell using Li@CC/Cu, with a cycling of 98 cycles prior to the cell failure (**Figures S9**a and **S9**c). A stable cycling over 120 cycles was achieved by the cell using Li@CCG/Cu with stable charge-discharge profiles (**Figures S9**a and **S9**d), indicating a high Li utilization by the host of CCG/Cu.

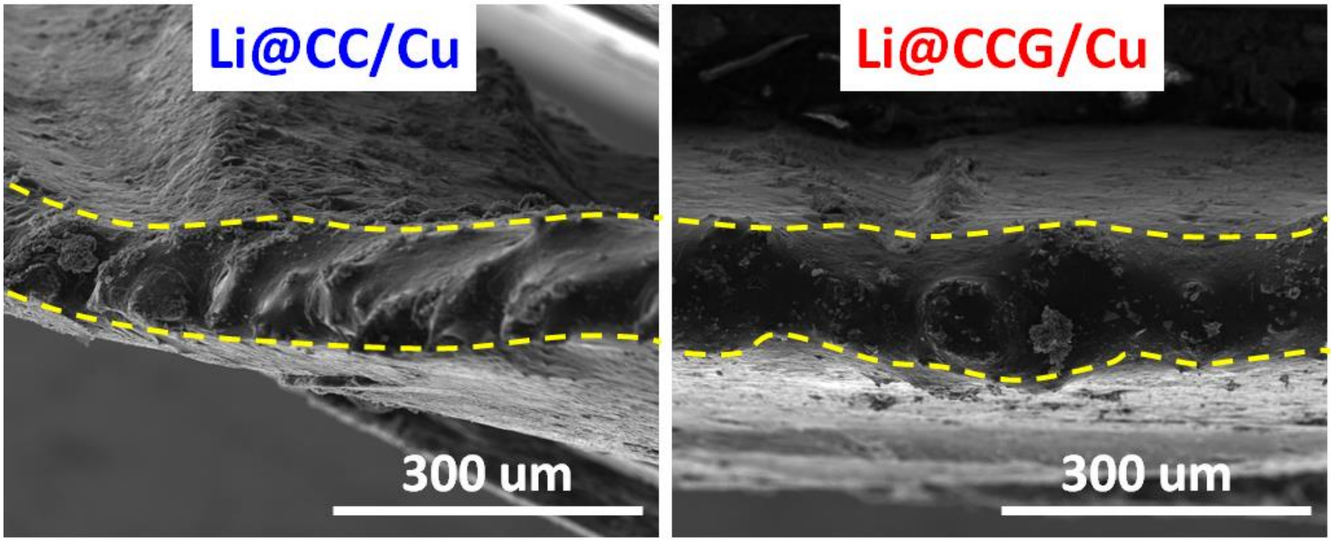

Figure S10. Cross-sectional SEM images of (a) Li@CC/Cu and (b) Li@CCG/Cu.

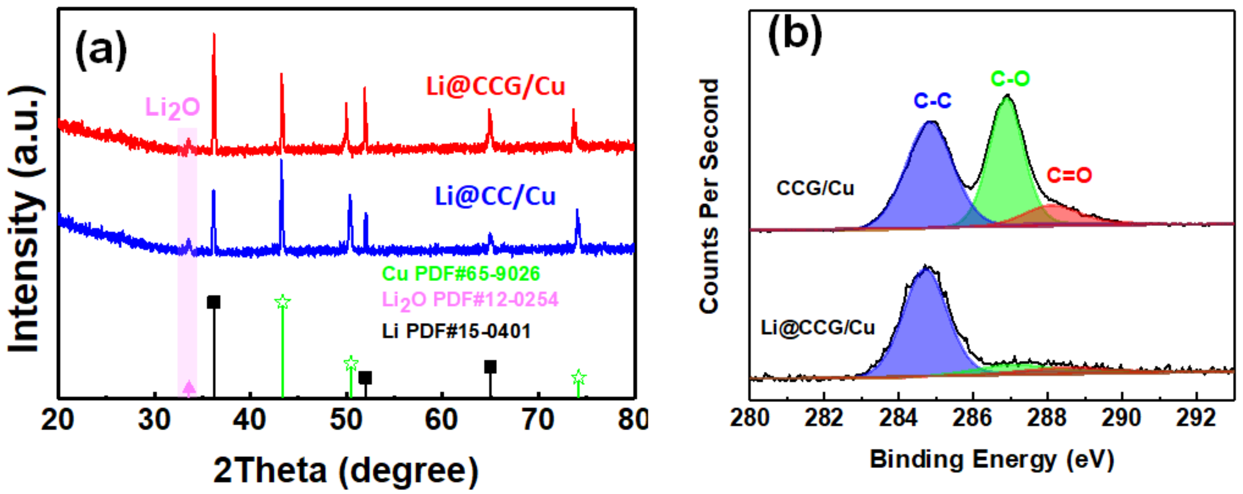

Figure S11. (a) XRD patterns of Li@CC/Cu and Li@CCG/Cu, (b) C 1s XPS spectra of CCG/Cu and Li@CCG/Cu.

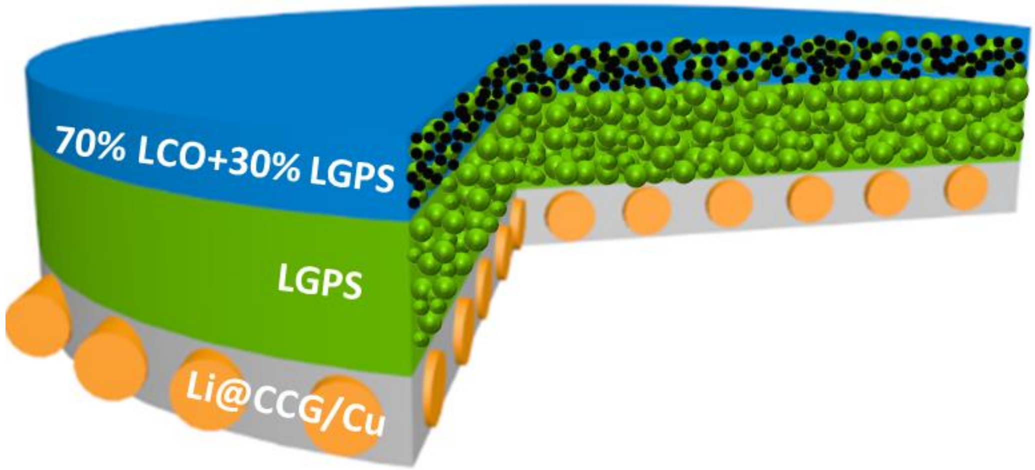

Figure S12. Schematic representation of the SSB using Li@CCG/Cu as anode, LCO as cathode and LGPS as electrolyte. Other SSBs were assembled with the same structure by only changing the anode.

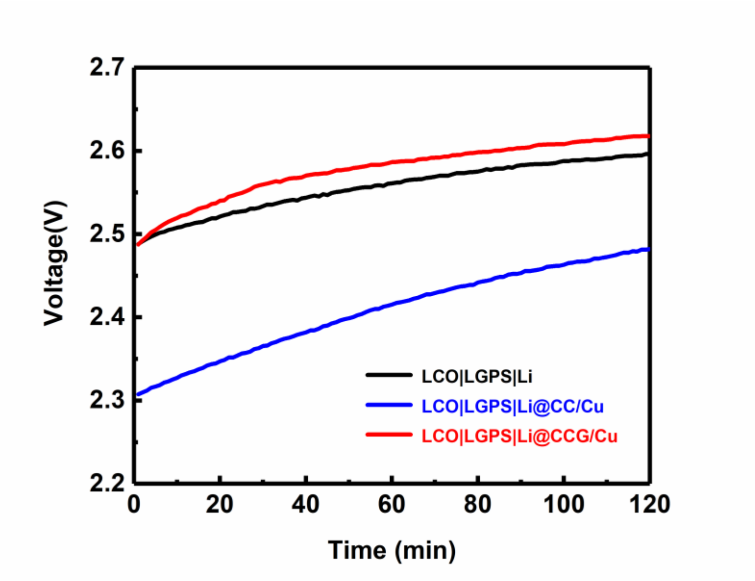

Figure S13. OCVs of the SSBs using Li, Li@CC/Cu and Li@CCG/Cu anodes.

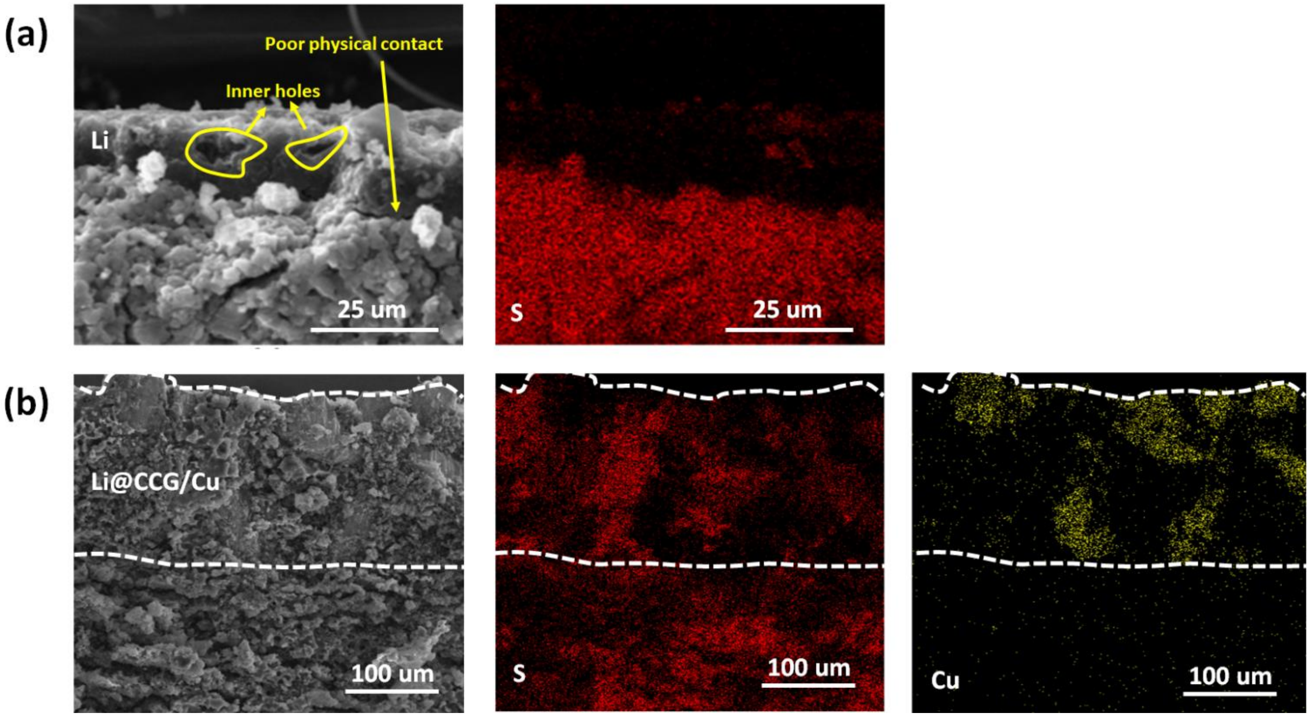

Figure S14. SEM images and EDS mapping of the anode/LGPS interfaces for the cycled cells using (a) Li and (b) Li@CCG/Cu anodes.

References

1. M. Wang, Z. Peng, W. Luo, F. Ren, Z. Li, Q. Zhang, H. He, C. Ouyang, D. Wang, Tailoring Lithium Deposition via an SEI-Functionalized Membrane Derived from LiF Decorated Layered Carbon Structure. *Adv. Energy Mater.* **2019**, 9, 1802912.
2. Y. Tao, S. Chen, D. Liu, G. Peng, X. Yao, X. Xu, Lithium Superionic Conducting Oxysulfide Solid Electrolyte with Excellent Stability against Lithium Metal for All-Solid-State Cells. *J. Electrochem. Soc.* **2015**, 163, A96-A101.
3. J. Yin, X. Yao, G. Peng, J. Yang, Z. Huang, D. Liu, Y. Tao, X. Xu, Influence of the Li-Ge-P-S based solid electrolytes on NCA electrochemical performances in all-solid-state lithium batteries. *Solid State Ionics* **2015**, 274, 8-11.
4. G. Kresse, J. Furthmuller, Efficient iterative schemes for ab initio total-energy calculations using a plane-wave basis set. *Phys. Rev. B* **1996**, 54, 11169-11186.
5. P. E. Blochl, Projector augmented-wave method. *Phys. Rev. B* **1994**, 50, 17953-17979.
6. J. P. Perdew, K. Burke, M. Ernzerhof, Generalized Gradient Approximation Made Simple. *Phys. Rev. Lett.* **1996**, 77, 3865-3868.
7. J. D. Pack, H. J. Monkhorst, "Special points for Brillouin-zone integrations"-a reply. *Phys. Rev. B* **1977**, 16, 1748-1749.
8. S. Grimme, J. Antony, S. Ehrlich, H. Krieg, A consistent and accurate ab initio parametrization of density functional dispersion correction (DFT-D) for the 94 elements H-Pu. *J. Chem. Phys.* **2010**, 132, 154104.
